# Subgroup analysis of imaging scans, invasive examinations and prognosis in mild-to-moderate isolated foetal cerebral ventriculomegaly: a retrospective study in China

**DOI:** 10.1177/03000605241301879

**Published:** 2024-12-09

**Authors:** Chun Yang, Xia Chi, Yan Wang, Cuiping Zhang, Ran Zhou, Xuemei Jia, Fengchang Qiao, Zhengfeng Xu

**Affiliations:** 1 Department of Prenatal Diagnosis, Women’s Hospital of Nanjing Medical University (Nanjing Women and Children’s Healthcare Hospital), Nanjing, China; 2 Department of Gynecology, Women’s Hospital of Nanjing Medical University (Nanjing Women and Children’s Healthcare Hospital), Nanjing, China; 3 Department of Obstetrics and Gynecology, Changzhou Second People’s Hospital Affiliated to Nanjing Medical University, Changzhou, China; 4 Department of Children Healthcare, Women’s Hospital of Nanjing Medical University (Nanjing Women and Children’s Healthcare Hospital), Nanjing, China; 5 Nanjing Medical Key Laboratory of Female Fertility Preservation and Restoration, Nanjing, China

**Keywords:** Ventriculomegaly, chromosomal aberration, malformation, cytomegalovirus infection, prognosis, brain, live birth

## Abstract

**Objective:**

This study aimed to analyse the causes of foetal mild-to-moderate isolated ventriculomegaly (IVM) and to evaluate the prognosis of neurological development in surviving children in different subgroups.

**Methods:**

We retrospectively studied mild-to-moderate IVM diagnosed by prenatal ultrasound scans in different subgroups according to the laterality of IVM, the degree of IVM and foetal sex independently. The results of foetal chromosomal microarray analysis, virological tests of umbilical cord blood or amniotic fluid, foetal magnetic resonance imaging and ultrasound were collected. Long-term follow-up was performed to assess the neurodevelopment of children within 66 months through telephone interviews and/or the Ages and Stages Questionnaire-3.

**Results:**

The moderate group showed more chromosomal abnormalities (16.2% vs. 4.1%) and greater structural anomalies in the brain (31.8% vs. 7.5%) than the mild group. Female foetuses showed more structural anomalies than male foetuses (25.0% vs. 7.2%). However, an adverse prognosis of children was not different across the different subgroups.

**Conclusion:**

Moderate IVM may be more strongly associated with chromosomal aberrations and structural malformations than mild IVM. However, the adverse prognosis of children was similar between the different subgroups analysed.

## Introduction

**Figure 1.**
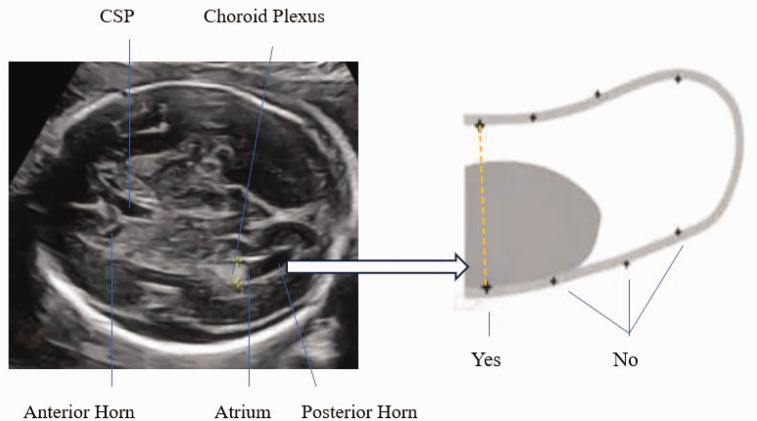
Schematic diagram of measurement of foetal lateral ventricles.

Foetal cerebral ventriculomegaly (VM) is identified as the width of the atrium of the lateral ventricles exceeding 10 mm (2.5–4 standard deviations above the mean) in the transventricular plane across the second and third trimesters of gestation.^1^ The transventricular plane shows the Falx, frontal horns, cavum septi pellucidi, choroid plexus, posterior horn, and symmetrical cerebral hemispheres^2^ (Figure 1). VM is relatively common in prenatal ultrasound findings, with an incidence of approximately 0.16% to 1%.^2,3^ If VM occurs, the most important course of action is to determine the underlying pathogenesis and evaluate the possible prognosis to help pregnant women make appropriate decisions. Multiple studies have shown that the prognosis of foetuses with severe VM (>15 mm) is poor. Severe VM is usually highly associated with chromosomal abnormalities, structural anomalies, intrauterine infections, or gene mutations.^4–9^ Additionally, the prognosis of patients with severe VM is unfavourable, and only approximately 42.2% of survivors have normal neurodevelopment.^10^ However, the pathogenesis and prognosis of foetuses with mild (10–12 mm) or moderate (12.1–14.9 mm) VM varies across studies.^5^ With the development of genetic diagnostic technology, more chromosomal abnormalities have been detected via chromosomal microarray analysis (CMA), and the CMA test is recommended as a diagnostic test for foetal VM by the Society for Maternal Foetal Medicine.^2^ However, only a limited number of studies and small case series have reported CMA, foetal magnetic resonance imaging (MRI), a virological test for intrauterine infection of toxoplasmosis, rubella, cytomegalovirus, and herpes simplex virus (TORCH) and the prognosis in foetuses with mild-to-moderate isolated ventriculomegaly (IVM) in the brain.^6,11^

In this study, we retrospectively assessed the incidence of chromosomal aberrations, intrauterine TORCH infection, and brain structural anomalies in foetuses with IVM who were diagnosed by an ultrasound (US) examination and who underwent a CMA test, foetal cytomegalovirus (CMV)-DNA detection or a TORCH-immunoglobulin M (IgM) assay, MRI, and dynamic US scans. Additionally, we followed the neurodevelopment of the newborns and compared the results of different subgroups.

## Methods

### Editorial policies and ethical considerations

This study was approved by the Ethics Committee of Maternity and Child Health Care Hospital affiliated with Nanjing Medical University (No. (2019) KY-081). All the women signed informed consent forms.

### Patient enrolment

Women with isolated mild-to-moderate foetal IVM from September 2015 to March 2021 were retrospectively recruited for this study. The mothers were diagnosed with foetal mild-to-moderate IVM by a US scan. All of the mothers were provided prenatal counselling by senior clinical genetic specialists and were rechecked by senior US specialists to confirm the presence of isolated mild-to-moderate VM in their foetuses at the Maternity and Child Health Care Hospital Affiliated with Nanjing Medical University. The inclusion criteria consisted of a preliminary diagnosis of mild-to-moderate IVM by US and the completion of laboratory tests in our hospital. The exclusion criteria were severe foetal VM, mild or moderate foetal VM with central nervous system (CNS) or extra-CNS anomalies diagnosed by US, instrument-assisted delivery (forceps or vacuum-cups), foetal hypoxia, neonatal asphyxia, neonatal pathological jaundice and missing data.

### CMA test and evaluation of copy number variants

The DNA of the foetuses was extracted from amniotic fluid, umbilical cord blood or foetal skin tissue. The DNA of the parents was obtained from peripheral blood at the same time. The QIAamp DNA Mini kit (Qiagen, Hilden, Germany) was used to extract DNA from the uncultured samples according to standard procedures. The DNA concentration and quality were detected via a NanoDrop spectrophotometer (Thermo Fisher, Waltham, MA, USA). DNA with median A260/280 absorbance ratios of 1.8 to 1.9 was selected and 250 ng of DNA was used according to the manufacturer’s instructions. The DNA was examined with an Affymetrix CytoScan 750K array (Affymetrix, Santa Clara, CA, USA), which includes 550,000 copy number variant (CNV) probes and 200,000 single nucleotide polymorphism (SNP) probes. CNVs larger than 100 kb or those that affected more than 50 contiguous probes were considered and annotated by the GRCh37 (hg19) genome. The identified CNVs were evaluated by studies and public databases such as DGV, RefSeq, UCSC, OMIM, Decipher, Gene Reviews, ClinVar and ClinGen, and PubMed, and were classified into five categories after evaluation: pathogenic (P) CNVs, likely pathogenic (LP) CNVs, variation of uncertain significance (VOUS), likely benign (LB) CNVs and benign (B) CNVs according to the American College of Medical Genetics and Genomics technical standards.^12,13^

### CMV-DNA detection and the TORCH-IgM assay

The detection of CMV-DNA was conducted using fluorescence quantitative polymerase chain reaction (Daan gene kit for DNA products; Daan Gene, Guangzhou, China) using a sample of amniotic fluid, which was mainly from foetal urine. The TORCH-IgM panel was performed using a chemiluminescent immunoassay (LIAISON1 TORCH IgM assay; CLIA; Liaison, Mirandola, Italy) using a sample of foetal blood obtained by umbilical cord blood puncture.

### Foetal MRI

Foetal MRI was performed to assess whether there were other subtle CNS structural malformations after 25 weeks of gestational age. We particularly focused on the third and fourth ventricles, midline falx, cavity of the septum pellucidum, corpus callosum, cisterna magna, thalami, germinal matrix region, cerebellum, and spine in addition to the lateral ventricles and choroid plexus according to the Society for Maternal Foetal Medicine recommendation.^2^

### US scans and follow-up

All of the pregnant women who had foetal cerebral lateral VM detected were examined by experienced senior prenatal US specialists. The lateral ventricle was measured in the transventricular (axial) plane at the level of the glomus of the choroid plexus and parietal–occipital groove, and we examined the frontal horns, cavum septum pellucidum, and symmetrical cerebral hemispheres. The sonographer positioned the callipers at the internal margin of the lateral and medial walls of the atria on an axis perpendicular to the long axis of the lateral ventricles and measured the atrial diameter. When the lateral ventricles were >10 mm, prenatal counselling was offered, and dynamic US was used to evaluate the progress of the width and to determine whether there were other anomalies of the CNS or extra-CNS every 2 to 3 weeks.

### Follow-up and neurodevelopment assessment

All of the patients were offered a telephone follow-up and/or to fill out the Ages and Stages Questionnaire-3 (ASQ-3) after their children were aged 6 to 66 months. The telephone follow-up consisted of the outcome of pregnancy, gestational week of delivery, birth date, mode of delivery, whether there was instrument-assisted delivery (forceps or a vacuum-cup), foetal hypoxia, neonatal asphyxia, neonatal pathological jaundice, and whether the language, motor, intelligence, communication and other abilities are age-appropriate.

The ASQ-3 consisted of questions about communication, fine motor, gross motor, personal–social, problem-solving, and total development at different time points from 6 to 66 months and 30 items specific to different age groups (6, 8, 12, 14, 16, 18, 20, 22, 24, 27, 30, 33, 36, 42, 48, 54, and 60 months). Each item includes three response options (yes, sometimes, and not yet), and each area of development is assigned a score between 0 and 60. The ASQ-3 provides a result of above, near, or below the critical value^14,15^ Children with scores near or below the critical value were clinically evaluated for neurodevelopment delay by a childcare physician and provided necessary rehabilitation guidance.

### Data collection and statistical analyses

The results of the CMA test, TORCH-IgM assay or CMV-DNA by polymerase chain reaction, foetal MRI scan, and US examination were collected from medical records through the Haitai outpatient management system and prenatal diagnosis database in our hospital. The outcome of the foetus and the neurodevelopmental condition of the child were determined by telephone interviews and/or the ASQ-3 at least once. The characteristics of ventriculomegalies, namely the laterality (unilateral or bilateral), degree (mild [10–12 mm] or moderate [12.1–15 mm]), and foetal sex (male or female), were recorded. Quantitative data are expressed as the mean ± standard deviation, and qualitative data are expressed as the number and percentage. Comparison between different categorical variables was performed using Fisher’s exact test. The data were analysed using IBM SPSS Statistics for Windows, Version 26 (IBM Corp., Armonk, NY, USA).

## Results

### Patients’ characteristics

**Figure 2.**
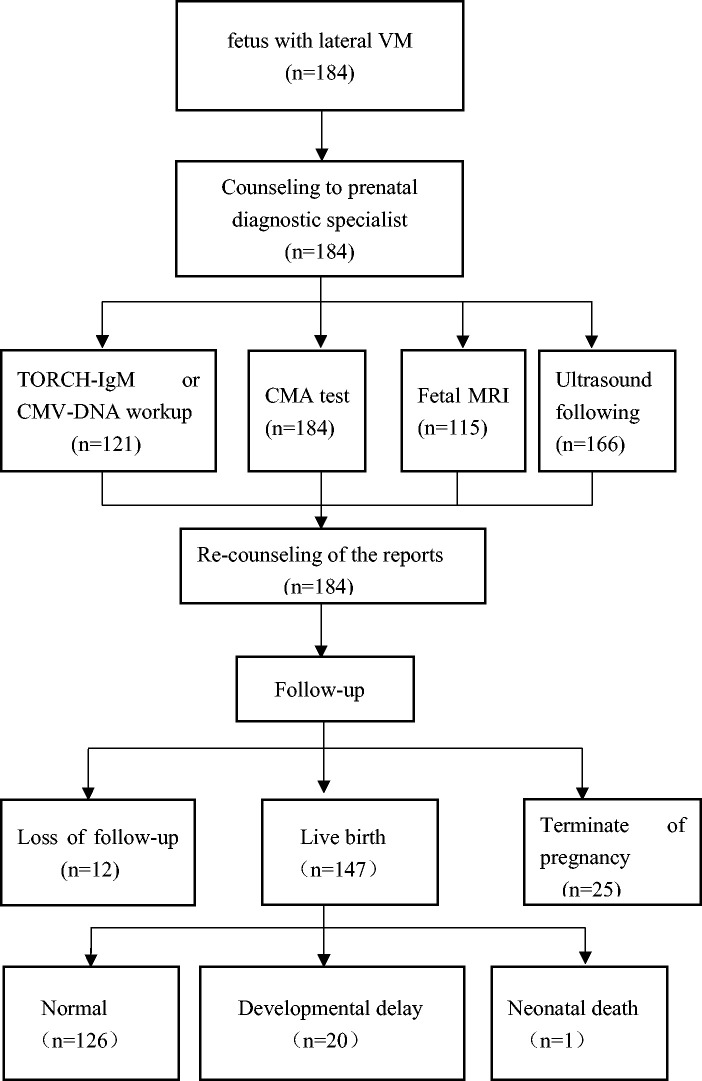
Flowchart of the study.

A total of 184 patients who met the inclusion criteria were identified and detected by the CMA. The mean gestational age was 26.15 ± 2.50 weeks and the mean maternal age was 29.05 ± 3.97 years. Among them, 121 patients underwent detection of CMV-DNA from amniotic fluid or TORCH-IgM from umbilical cord blood. Additionally, 115 patients had foetal MRI performed to determine whether there were structural abnormalities of the brain after 25 gestational weeks. Sixty-six patients were followed up by a US examination, with a focus on the progression of foetal VM and the appearance of CNS or extra-CNS anomalies every 2 or 3 weeks. Twelve patients were lost to follow-up, and 25 patients decided to terminate the pregnancy. A total of 147 patients had live births, and 126 children had normal neurodevelopment during the follow-up. However, 20 children suffered from developmental delay or other adverse outcomes and 1 newborn died within 3 days after birth (Figure 2).

### CNVs of the CMA

Using the CMA test, 125 CNVs in 80 (43.5%) foetuses were examined. Among them, 14 CNVs in 12 (6.5%) foetuses were identified as P/LP (2 of them were complicated by VOUS), 108 CNVs in 67 (36.4%) foetuses were identified as VOUS or ROH (2 of them were complicated by P/LP CNVs) and 3 CNVs in 3 (1.6%) foetuses were identified as B/LB. A total of 104 (56.5%) foetuses were normal (Table 1). A comparison of the different subgroups of IVM showed a significantly greater incidence of P/LP CNVs in the moderate group than in the mild group (P = 0.021). There was no significant difference in CNVs between the sexes or the degree of unilateral and bilateral VM (Table 1).

**Table 1.** Results of the CMA test in 184 foetuses with mild-to-moderate IVM.

Characteristics	Details	P/LP CNV, n (%)	VOUS CNV, n (%)	Normal+ B/LB CNV, n (%)	Total, n	P value
Laterality of IVM	Unilateral	5 (4.4)	39 (33.9)	71 (61.7)	115	0.217
	Bilateral	7 (10.1)	26 (37.7)	36 (52.2)	69	
Degree of IVM	Mild	6 (4.1)	55 (37.4)	86 (58.5)	147	0.021
	Moderate	6 (16.2)	10 (27.0)	21 (56.8)	37	
Foetal sex	Male	7 (5.4)	44 (33.8)	79 (60.8)	130	0.521
	Female	5 (9.3)	21 (38.9)	28 (51.8)	54	
Total		12 (6.5)	65 (35.3)	104 + 3 (58.2)	184	

CMA, chromosomal microarray analysis; IVM, isolated ventriculomegaly, P, pathogenic; LP, likely pathogenic; CNV, copy number variant; VOUS, variant of uncertain significance; B, benign; LB, likely benign.

### Congenital intrauterine infection of TORCH

Only 1.7% (2/121) of foetuses were diagnosed with intrauterine CMV infection, both of whom presented with mild IVM. There were no significant differences between the different subgroups (Table 2).

**Table 2.** Results of CMV-DNA detection or the TORCH-IgM assay in amniotic fluid or umbilical cord blood of 121 foetuses with mild-to-moderate IVM.

Characteristics	Details	Positive, n (%)	Negative, n (%)	Total, n	P value
Laterality of IVM	Unilateral	1 (1.4)	73 (98.6)	74	0.745
	Bilateral	1 (2.1)	46 (97.9)	47	
Degree of IVM	Mild	2 (2.0)	99 (98)	101	0.527
	Moderate	0 (0)	20 (100)	20	
Foetal sex	Male	2 (2.2)	87 (97.8)	89	0.394
	Female	0 (0)	32 (100)	32	
Total		2 (1.7)	119 (98.3)	121	

CMV-DNA, cytomegalovirus-DNA; TORCH-IgM, immunoglobin M of toxoplasmosis, rubella virus, cytomegalovirus, and herpes virus; IVM, isolated ventriculomegaly.

### Brain structural abnormalities detected by foetal MRI

A total of 12.2% (14/115) of patients were complicated by one or more type of foetal brain structural abnormalities as shown by MRI. There were eight (7.0%) foetuses with hypoplasia or the absence of the corpus callosum, four (3.5%) with intracranial cysts and seven (6.1%) who progressed to severe VM (Table 1s). A subgroup analysis showed that the incidence of cerebral structural anomalies on foetal MRI was significantly greater in the moderate group than in the mild group (P = 0.006) and in the female group than in the male group (P = 0.022). However, the incidence of cerebral structural anomalies on foetal MRI was not significantly different between the bilateral and unilateral groups (Table 3).

**Table 3.** Incidence of cerebral structural anomalies as shown by foetal magnetic resonance imaging in 115 foetuses with mild-to-moderate IVM.

Characteristics	Details	Positive, n (%)	Negative, n (%)	Total, n	P value
Laterality of IVM	Unilateral	6 (8.6)	64 (91.4)	70	0.141
	Bilateral	8 (17.8)	37 (82.2)	45	
Degree of IVM	Mild	7 (7.5)	86 (92.5)	93	0.006
	Moderate	7 (31.8)	15 (68.2)	22	
Foetal sex	Male	6 (7.2)	77 (92.8)	83	0.022
	Female	8 (25.0)	24 (75.0)	32	
Total		14 (12.2)	101 (87.8)	115	

IVM, isolated ventriculomegaly.

### Results of US follow-up

A total of 6.6% (11/166) of patients had one or more than one type of foetal CNS or extra-CNS structural abnormality as shown by a dynamic US examination follow-up after being first diagnosed with mild-to-moderate foetal IVM every 2 to 3 weeks. Four patients had foetal IVM that progressed beyond 15 mm, three did not have a corpus callosum, one had an intracranial cyst, one had enlargement of the posterior fossa >15 mm, two had severe hydronephrosis, one was complicated by short long bones and one had intestinal dilatation. There were no significant differences in the laterality of IVM, the degree of IVM, or foetal sex between the different subgroups (Table 4 and Table 1s).

**Table 4.** Ultrasound follow-up of 166 foetuses with mild-to-moderate IVM.

Characteristics	Details	Positive, n (%)	Negative, n (%)	Total, n	P value
Laterality of IVM	Unilateral	8 (7.2)	103 (92.8)	111	0.924
	Bilateral	3 (5.5)	52 (94.5)	55	
Degree of IVM	Mild	8 (6.0)	126 (94)	134	0.764
	Moderate	3 (9.4)	29 (90.6)	32	
Foetal sex	Male	7 (6.0)	110 (94)	117	0.863
	Female	4 (8.2)	45 (91.8)	49	
Total		11 (6.6)	155 (93.4)	166	

IVM, isolated ventriculomegaly.

### Outcomes of pregnancies and neurodevelopmental assessment of survivors

A total of 6.5% (12/184) of patients were lost to follow-up. A total of 14.5% (25/172) of gravidas chose termination of pregnancy (9 cases of P/LP CNVs, 2 patients with intrauterine infection of CMV, and 14 patients with foetal CNS or extra-CNS structural anomalies) (Table 1s). Additionally, 85.47% (147/172) of patients had live births. The patients had a telephone follow-up and/or filled out the ASQ-3 when their children were aged 37.75 ± 18.72 months after birth. Among them, 14.3% (21/147) of children had different types of adverse outcomes (13 children had a mild developmental speech or language or gross motor disorder, 6 had global neurodevelopment delay, 1 had epilepsy, and there was 1 unexplained neonatal death 3 days after birth) (Figure 2, Table 1s). The six children with global neurodevelopment delay and the child with epilepsy were classified as having severe adverse outcomes. The incidence of total adverse outcomes or severe adverse outcomes was not associated with the three subgroups (Table 5).

**Table 5.** Adverse outcomes and severe adverse outcomes of 147 children who were diagnosed with mild-to-moderate IVM prenatally.

Characteristics	Details	Total, n	Adverse outcome n (%)	Normal, n (%)	P value	Severe adverse outcome	Normal or mild NDD	P value
Laterality of IVM	Unilateral	98	13 (13.3)	85 (86.7)	0.617	4 (4.1)	94 (95.9)	0.585
	Bilateral	49	8 (16.3)	41 (83.7)		3 (6.1)	46 (93.9)	
Degree of IVM	Mild	119	16 (13.4)	103 (86.6)	0.941	4 (3.4)	115 (96.6)	0.250
	Moderate	28	5 (17.9)	23 (82.1)		3 (10.7)	25 (89.3)	
Foetal sex	Male	105	14 (13.3)	91 (86.7)	0.602	3 (2.9)	102 (97.1)	0.198
	Female	42	7 (16.7)	35 (83.3)		4 (9.5)	38 (90.5)	
Total		147	21 (14.3)	126 (85.7)		7 (4.8)	140 (95.2)	

IVM, isolated ventriculomegaly; NDD, neurodevelopment delay.

## Discussion

Foetal cerebral lateral VM is an important US soft marker of the foetal nervous system. Previous studies have shown that the causes of VM include chromosomal abnormalities, intrauterine infection of TORCH, intracranial structural anomalies, and other unknown reasons.^5,9–11^ Multiple studies have compared the incidence of these abnormalities between IVM and non-IVM, between mild-to-moderate VM and severe VM, and between bilateral VM and unilateral VM.^5,9–11^ However, few studies have compared the incidence of these abnormalities and the outcomes of live births with a focus on isolated mild-to-moderate VM and different subgroups.^6^

CMA is recommended to determine foetal ventriculomegaly because cerebral lateral VM is associated with chromosomal abnormalities. However, the incidence of pathogenic or likely pathogenic CNVs in VM varies from 3.6% to 16%,^6,11,16–20^ and whether an invasive prenatal diagnostic test or MRI should be offered to gravidas with isolated mild or moderate foetal VM is controversial.^17–22^ Our study showed that the percentage of P/LP CNVs was 6.5% (12/184) in gravidas with mild (135 cases) to moderate (37 cases) foetal IVM. This finding is consistent with that by Wang et al. (6.51%)^5^ and He et al. (6.2%),^16^ and a meta-analysis showed that the incidence of chromosomal abnormalities was 9% with a 95% confidence interval of 4% to 16% in mild VM.^11^ To evaluate the contribution of the CMA test to comparing different subgroups of mild-to-moderate foetal IVM, we performed a subgroup analysis of 184 women. We found that foetuses with bilateral IVM, foetuses with moderate IVM and female foetuses showed a greater incidence of P/LP CNVs than those with unilateral IVM, those with mild IVM and male foetuses. However, there was a significant difference only between the mild and moderate IVM groups. No studies have compared the incidence of CNVs between mild IVM and moderate IVM. Our results are in disagreement with those in other studies in China. A previous study showed that the incidence of chromosomal abnormalities was much greater in the bilateral group than in the unilateral group (14.3% vs. 4.1%, P = 0.011).^17^ However, this previous study took severe IVM into account, and severe VM is more likely to be bilateral. Additionally, the authors of this previous study did not compare mild and moderate VM, and only compared mild-to-moderate VM and severe VM. Wang et al. showed a much greater incidence of chromosomal abnormalities in the bilateral group than in the unilateral group (10.56% vs. 5.71%, P = 0.04) and found a similar incidence in the mild and moderate groups (5.92% vs. 9.63%, P = 0.208).^5^ However, their study included foetuses with isolated and non-isolated VM from mild to severe cases. Our study suggested that foetuses with mild-to-moderate IVM should be diagnosed by CMA, especially those with moderate IVM, regardless of whether they are unilateral or bilateral, or male or female foetuses.

The incidence of intrauterine infection of TORCH in our study was 1.7% (2/121), and both of the foetuses were characterised as mild IVM. This result is consistent with that of Devaseelan et al. (1.5%),^23^ but lower than that of a meta-analysis in which the incidence of TORCH infection was 8.2% (95% confidence interval, 3.6%–14.5%).^24^ The reason for this discrepancy between this meta-analysis and our study is that, in the meta-analysis,^24^ the authors took into account five cases of maternal CMV infection, but in fact, only one case was verified as foetal congenital infection after birth.^25^ Therefore, intrauterine infection of TORCH should be confirmed by samples obtained from amniocentesis or umbilical cord blood puncture, not with samples of maternal blood. Another study reported that one patient was diagnosed with congenital infection but the sample size was small (only 15 patients).^26^ We compared different subgroups and found that there was no significant association between the laterality of IVM, the degree of IVM, or foetal sex and intrauterine infection of TORCH. However, there may have been bias because the number of positive cases was small.

Foetal MRI has the advantage of facilitating analysis by enabling flexible adjustment of imaging planes to achieve optimal visualisation and does not harm the foetus. MRI has been increasingly applied to diagnose foetal CNS malformations in recent years.^27–31^ MRI is the imaging method of choice for observing foetal CNS structure, followed by a US scan. In our study, the overall prevalence of additional CNS anomalies detected by foetal MRI was 12.2% (14/115), which is consistent with most other previous reports^24,32,33^ but higher than that reported by Wang et al^5^ in another centre in China (3.54%, 18/509). These results suggest that foetal MRI increases the detection rate of CNS anomalies for mild IVM diagnosed by a US examination by 3.5% to 10%. Among the 14 patients with foetal intracranial abnormalities identified by MRI, 1 had pathogenic CNV, and 6 also had a US review. The remaining seven cases of mild-to-moderate foetal IVM were found only by MRI, indicating that the percentage of CNS structural anomalies (e.g., agenesis of the corpus callosum, an intracranial cyst, and dysplasia of the cerebral parenchyma) was 6.1%, which is consistent with the results of the ENSO Working Group (5.4%).^31^ Therefore, foetal MRI plays an important role in the diagnosis of VM and other CNS anomalies. Additionally, the subgroup analysis showed that the number of positive MRI findings was significantly greater in the moderate group than in the mild group and significantly greater in the female group than in the male group, but only tended to be greater in the bilateral group than in the unilateral group. Therefore, foetal MRI should be carried out for all foetuses with mild-to-moderate cerebral IVM, especially for those with moderate IVM and female foetuses. MRI cannot be replaced by a US examination because US scans have difficulty demonstrating parts of brain structures, such as the corpus callosum and other subtle anomalies.

A US examination can provide clear images of most foetal organ structures and be used to identify most structural abnormalities. A routine US examination and systematic US screening have been incorporated into prenatal screening and diagnosis practice guidelines worldwide. A large study in the UK showed that the overall diagnostic accuracy of US for VM was 89.9%.^27,34^ A US examination often has difficulty in identifying some intracranial structures, such as the corpus callosum and intracranial cysts, because the display requires scanning at the axial, coronal, and sagittal planes of the foetal brain.^35^ However, US is convenient for a dynamic review of the ventricular width, and CNS or extra-CNS malformations appear several weeks after mild-to-moderate IVM. In our study, 184 patients had mild-to-moderate foetal IVM as shown by a US scan, and 166 had dynamic US follow-up. Eleven (6.6%) cases progressed to severe VM, other intracranial structural abnormalities or extra-CNS abnormalities. Among them, six patients also showed foetal intracranial structural anomalies on MRI. In addition, among the remaining five patients, one showed aggravated enlargement of the ventricles with posterior fossa dilation, one had absence of the corpus callosum, one had progression to severe VM, and two patients presented with extra-CNS abnormalities (one patient with severe hydronephrosis and one patient with short long bones) by dynamic US follow-up. Previous studies showed that 7% to 12.8% of ongoing pregnancies with mild VM were associated with major abnormalities on a US examination, which cannot be found in the initial scan.^33,36^ This is consistent with our results. Additionally, the five patients mentioned above had no pathogenic findings in foetal brain MRI, the TORCH test or CMA, which indicated that dynamic US contributed to an additional 3.0% (5/166) of CNS or extra-CNS structural anomalies. Therefore, US follow-up is essential for foetuses with mild-to-moderate IVM even if there are no positive findings by an invasive prenatal diagnosis or MRI. Further analysis showed that the incidence of US malformations was not significantly different among the different subgroups. Therefore, US should be conducted on all cases of mild-to-moderate foetal IVM to assess its progress and to identify the appearance of CNS and extra-CNS structural malformations.

Through telephone follow-up and/or ASQ evaluation, we found that the incidence of abnormal neurodevelopment of children in our study was 14.3% (21/147). Among the children, 4.1% (6/147) had global developmental disorders, 0.1% (1/147) had epilepsy, 0.1% (1/147) had unexplained neonatal death 3 days after birth, 5.4% (8/147) had mild speech developmental delay, 2.0% (3/147) had mild motor developmental delay, and 1.4% (2/147) had ocular dysfunction. Our finding of the incidence of abnormal neurodevelopment is similar to that (8.3%) in foetuses with US soft markers excluding chromosomal aberrations^20^ and is higher than that (2.2%) in a reference population.^37^ The subgroup analysis showed that adverse outcomes of foetuses with mild-to-moderate IVM who survived were not associated with the laterality of IVM, the degree of IVM, or foetal sex. These results indicate that more attention should be paid to mild-to-moderate IVM, and more research needs to be performed to investigate the reasons for abnormal neurodevelopment.

## Conclusions

The CMA test, TORCH panel, and imaging scans by US or MRI contribute to the diagnosis and aetiological identification of gravidas with foetal mild-to-moderate IVM, and any of the approaches cannot be used instead of others. Moderate IVM may be more strongly associated with chromosomal aberrations and structural malformations than mild IVM. However, the adverse prognosis of children after a prenatal diagnosis is not associated with the foetal sex or laterality or degree of foetal IVM. Our study may help in genetic counselling in clinical practice for these women.

There are still several limitations to this study. First, this was a single-centre study, and the number of cases included was small. Second, some children with mild-to-moderate IVM still showed unexplained neurodevelopmental delay. Therefore, further investigation of the aetiology of neurodevelopment delay without chromosomal abnormalities, intrauterine infection or intracranial structural abnormalities in these children is required.

## Data Availability

All data are available on request from the corresponding author.
